# An Approach Using Emerging Optical Technologies and Artificial Intelligence Brings New Markers to Evaluate Peanut Seed Quality

**DOI:** 10.3389/fpls.2022.849986

**Published:** 2022-04-14

**Authors:** Gustavo Roberto Fonseca de Oliveira, Clíssia Barboza Mastrangelo, Welinton Yoshio Hirai, Thiago Barbosa Batista, Julia Marconato Sudki, Ana Carolina Picinini Petronilio, Carlos Alexandre Costa Crusciol, Edvaldo Aparecido Amaral da Silva

**Affiliations:** ^1^Department of Crop Science, College of Agricultural Sciences, São Paulo State University, Botucatu, Brazil; ^2^Laboratory of Radiobiology and Environment, Center for Nuclear Energy in Agriculture, University of São Paulo, Piracicaba, Brazil; ^3^Department of Exacts Sciences, College of Agriculture “Luiz de Queiroz”, University of São Paulo, Piracicaba, Brazil

**Keywords:** *Arachis hypogaea* L., multispectral, images, machine-learning, fluorescence, reflectance, seed quality

## Abstract

Seeds of high physiological quality are defined by their superior germination capacity and uniform seedling establishment. Here, it was investigated whether multispectral images combined with machine learning models can efficiently categorize the quality of peanut seedlots. The seed quality from seven lots was assessed traditionally (seed weight, water content, germination, and vigor) and by multispectral images (area, length, width, brightness, chlorophyll fluorescence, anthocyanin, and reflectance: 365 to 970 nm). Seedlings from the seeds of each lot were evaluated for their photosynthetic capacity (fluorescence and chlorophyll index, F_0_, F_m_, and F_v_/F_m_) and stress indices (anthocyanin and NDVI). Artificial intelligence features (QDA method) applied to the data extracted from the seed images categorized lots with high and low quality. Higher levels of anthocyanin were found in the leaves of seedlings from low quality seeds. Therefore, this information is promising since the initial behavior of the seedlings reflected the quality of the seeds. The existence of new markers that effectively screen peanut seed quality was confirmed. The combination of physical properties (area, length, width, and coat brightness), pigments (chlorophyll fluorescence and anthocyanin), and light reflectance (660, 690, and 780 nm), is highly efficient to identify peanut seedlots with superior quality (98% accuracy).

## Introduction

Peanut (*Arachis hypogaea* L.) is an oleaginous crop with considerable relevance in agriculture (Stalker and Wilson, 2016). Nations such as China, India, Nigeria and the United States produce most of the peanuts consumed in the world and contribute to global food security (Stalker and Wilson, 2016; USDA, 2020b). Peanut seeds are rich in oil and proteins (Arya et al., 2016), in addition to chemical properties that play an essential role in human health and in combating malnutrition (Temba et al., 2016; Bessada et al., 2019). Considering that the peanut production chain spans over six continents (USDA, 2020a), exploring factors that favor grain yield is part of a comprehensive global food security strategy. Taking this strategy into account, post-harvest technologies can increase seed quality which in turn would represent an increased grain yield.

Seeds of high physiological quality are the basic input for agriculture. They have high vigor which means better ability to promote rapid crop establishment under wide environmental conditions with a direct contribution to plant establishment and yield (Finch-Savage and Bassel, 2016; Ebone et al., 2020). Seeds with high quality have a prolonged lifespan, which ensures the retention of their vigor until sowing (Sano et al., 2016; Basso et al., 2018). Due to factors such as harvest immaturity (Okada et al., 2021), mechanical damage in processing (Barbosa et al., 2014), storage fungi (Ding et al., 2015) and inadequate transportation conditions (Groot et al., 2022), peanut seeds lose their quality in the production process. Few studies provide solutions to maximize peanut seed quality at post-harvest. For other species of agricultural interest, non-destructive technologies that generate data from multispectral images have been successfully used to assess seed quality (Elmasry et al., 2019a; Mortensen et al., 2021). Considering this possibility, the peanut seed may present unexplored spectral markers that allow the efficient evaluation of this quality.

The possibility of evaluating seed quality through multispectral images has been shown for legumes such as soybean (Baek et al., 2019), cowpea (Elmasry et al., 2019b) and six other species (Hu et al., 2020). In the case of crops such as tomatoes and carrots (Galletti et al., 2020), low seed reflectance at short wavelengths and reduced chlorophyll fluorescence were identified as markers of their quality. Reflectance makes it possible to investigate the spectral behavior of plant tissues through the pattern of reflected light at different wavelengths (Meireles et al., 2020). The light reflectance properties are also affected by the physiological state of the plants under unfavorable conditions, such as water stress (Caturegli et al., 2020). The application of reflectance in seed studies allows the evaluation of fungal incidence (França-Silva et al., 2020; Rego et al., 2020), color (Wang X. et al., 2021) and chemical composition variations (Barboza da Silva et al., 2021a; Bianchini et al., 2021). Under another principle, fluorescence is detected by the excitation of chlorophylls (a/b) in plant tissues in specific bands of the spectrum (Murchie and Lawson, 2013). The dynamics of chlorophyll fluorescence in the seed domain may be associated with its maturity (Galletti et al., 2020) or aging (Barboza da Silva et al., 2021b). In the seedling domain, on the other hand, chlorophyll fluorescence behavior has to do with photosynthetic functioning (Herritt et al., 2020; Oliveira et al., 2021). Thus, peanut seeds and seedlings may present characteristics that can be useful to the seed industry.

With the development of data processing capacity, machine-learning algorithms are promising tools to autonomously categorize seedlot quality. This approach has been explored to identify seed patterns associated with physical, physiological, and health characteristics with high accuracy (Medeiros et al., 2020b; Barboza da Silva et al., 2021b; Bianchini et al., 2021). This approach has also been employed for seed variety identification (Taheri-Garavand et al., 2021b). In different species, the combination of multispectral images and algorithms has been highly effective for seed evaluation (Elmasry et al., 2019b; Hu et al., 2020). The idea of this research is that peanut seeds have markers of their quality which are detectable by these technologies. Here, it was investigated whether multispectral images combined with machine learning models can efficiently categorize the quality of peanut seedlots.

## Materials and Methods

### Plant Material

Seven lots of peanut (*Arachis hypogaea* L.; cv. IAC OL3; Virginia group) seeds produced in 2019/2020 in the western region of the State of São Paulo, Brazil by COPERCANA^1^ and COPLANA^2^ seed companies, were used for the research. The fruits were harvested and then dried in the shade. After this, the seeds were manually extracted. The seeds obtained from each lot were homogenized by manually removing broken or malformed seeds (sectioned or damaged cotyledons) and seeds without the tegument. The seedlots were stored in a dry chamber at 12°C/55% relative humidity (RH) until the beginning of the experiments, after approximately 90 days of storage.

### Trial Design

Initially, conventional tests were conducted to assess the quality of seedlots through water content, fresh weight, germination, and vigor. Then, from a study using multispectral images, it was found that certain spectral characteristics of the seeds correlated strongly with their quality. From the characteristics found through these images, the quality of seedlots was classified (principal component analysis) into groups of low vigor (lots 1, 2, and 3) and high vigor (lots 4, 5, 6, and 7). With this qualitative information (two groups), machine learning models (quadratic discriminant analysis method) were used to autonomously recognize these behaviors (high and low vigor). Finally, seedlings from the seeds in each lot were evaluated for their photosynthetic capacity and stress indicators using multispectral images. In addition, two other studies were conducted with seeds exposed to stress conditions (high temperature and high RH). Seedlings from these seeds were also evaluated for their photosynthetic capacity and stress indicators. Details regarding the variables measured, method, and number of seeds used in each research test are available for consultation in the supplementary files (Supplementary Tables 1, 2).

### Characterization of Physiological Quality of Seeds

The water content of the seeds was determined by the oven method at 105 ± 3°C for 24 h (ISTA, 2020), using four replicates of 10 seeds. For the determination of seed fresh weight, four replicates of 100 seeds were weighed on an analytical scale with a precision of 0.001 g. Subsequently, a part of the seeds of each lot (about 500 g) was treated with fungicides (Carbendazim and Thiram; 2 mL kg^–1^). This procedure aimed to inhibit the occurrence of fungi during the execution of the tests and to reduce any interference of pathogenic microorganisms in the seed quality results. The remaining seeds were not submitted to the treatment with fungicides. It was considered that any product applied to the surface of the seeds could change their spectral characteristics and compromise the quality of the data generated.

Germination was evaluated on rolled paper towel and sand substrates. Four replicates of 25 seeds were placed between the paper towels and moistened with deionized water at 2.5 times the mass of the dry paper. The rolled paper towels were kept at a constant 25°C in the dark. For the sand substrate, a sterile medium textured sand in plastic boxes was used (34.0 × 21.7 × 7.0 cm), and the substrate was wet to 60% of its holding capacity. Then, four replicates of 25 seeds from each lot were sown at a depth of 5.0 cm. The boxes with the seeds remained in a growth chamber at 25°C and 80% RH. The percentage of normal seedlings (with all their essential structures, such as aerial part, hypocotyl and well-developed radicle, complete, proportional and healthy) produced in the germination test using paper towels and sand was obtained on the 10th day (final score) after initial sowing (ISTA, 2020).

Vigor was initially determined by the time required for 50% germination (t50). Four replicates of 25 seeds from each lot were used according to the conditions described for the germination experiment between rolled paper towels. Twenty-four hours after the beginning of the experiment germination was assessed, with radicles with ≥2 mm in length used as the criteria. The measurements were performed every 4 h. The calculation of t50 was performed using the Germinator software (Joosen et al., 2010).

The seeds of each lot were also evaluated for seedling emergence capacity. Four replicates of 25 seeds each were used, with sand as substrate for the test. The seeds were sown at a depth of 5.0 cm in a suspended bed under uncontrolled environmental conditions. The substrate was wetted after sowing and throughout the experiment. Emerged seedlings (cotyledons and epicotyl apparent on the substrate surface) were counted daily and at the same time until stabilization of the number of emerged seedlings (Krzyzanowski et al., 2020). Seed vigor was expressed as percentage of emerged seedlings.

Another vigor test was carried out based on the seedling performance. For that, four replicates of 10 seeds were used, sown equidistantly from each other on the upper third of the surface of paper towels, using the same conditions described for germination between rolled paper towels. After 5 days, shoot and radicle length of normal seedlings was measured. Afterward, the aerial part and the radicles were segmented and placed in an oven at 60°C for 72 h to assess the dry weight (Krzyzanowski et al., 2020).

### Multispectral Image Acquisition of Seeds

Multispectral images were acquired from a total of 170 seeds for each lot. The seeds were placed in 9.0 cm glass Petri dishes. Multispectral images were captured at 19 wavelengths – 365 (UV), 405 (violet), 430 (indigo), 450 (blue), 470 (blue), 490 (cyan), 515 (green), 540 (green), 570 (yellow), 590 (amber), 630 (red), 645 (red), 660 (red), 690 (dark red), 780 (dark red), 850, 880, 940, and 970 nm (the last four wavelengths in the near infrared region), using a VideometerLab4™ instrument (Videometer A/S, Herlev, Denmark; software version 3.14.9) as described by Galletti et al. (2020). This system can capture and combine high-resolution multispectral images (2192 × 2192 pixels). Before acquiring the seed images, the light configuration was adjusted to optimize the intensity at each bandwidth, resulting in a better signal-to-noise ratio so that the captured images could be directly comparable. The light configuration was adjusted using a representative sample, and then the strobe time of each type of illumination was optimized in relation to this area. Seeds were segmented based on thresholding and the following variables were extracted from individual seeds: area, length, width and brightness measured by CIELab L* (Oliveira et al., 2021), fluorescence of chlorophyll *a* (630/700 nm excitation/emission) and chlorophyll *b* (405/600 nm excitation/emission). In addition, the reflectance values of the seeds of each lot from 365 to 970 nm were collected, and the chlorophyll *a/b* ratio was calculated. The seed images were transformed by a normalized canonical discriminant analysis (nCDA) algorithm, in which pixel values are calculated based on 10% trimmed mean to provide a more realistic image.

Multispectral images were also captured using a SeedReporter™ instrument (PhenoVation B.V., Wageningen, Netherlands) to calculate the anthocyanin index of the seeds. Prior to image acquisition, light intensity was adjusted to avoid overload. Reflectance images were acquired in a few seconds, generating multispectral images with a spatial dimension of 2448 × 2448 pixels (3.69 μm/pixel). A broad-band blank white light (3000 K) in a range of 450 to 780 nm was used to illuminate the seeds, and reflectance data was collected using three optical filters at 540, 710, and 770 nm (Gitelson et al., 2009). The anthocyanin index was calculated by SeedReporter™ software version 5.5.1. using the equation presented by Oliveira et al. (2021).

### Machine Learning – Quadratic Discriminant Analysis

The Quadratic Discriminant Analysis (QDA) method was used for the classification of high and low vigor seedlots. The choice of this method was based on the following aspects: (i) QDA is one of the most widely used methodologies for cases where the response variable is qualitative (Hastie et al., 2009; James et al., 2021) and (ii) it allows for effective analyses with data that do not have a normal distribution and have inhomogeneous variance and a covariance matrix structure (Clarke et al., 1979). Classification modeling was used based on the dataset extracted from the multispectral images of the seeds. Four QDA-based method machine learning models were generated for different datasets. In this way, the capacity of these models to infer the accuracy (sensitivity and specificity) of the spectral variables regarding the vigor of the seedlots (*n* = 1190) was tested. The learning models obtained through the QDA method were adjusted and tested by cross-validation using data related to the physical optical descriptors of the seedlots (first model: area, length, width and CIELab L*), pigments (second model: chlorophyll fluorescence and anthocyanins), reflectance (third model - bands that best discriminated seedlots: 660, 690, 780, 850, and 970 nm) and the sum of all these variables (fourth model: physical optical descriptors, pigments and reflectance). In all, four prediction models were built, and the data were divided into 70% for training and 30% for testing. The details of the mathematical procedures used are described in a supplementary file (Supplementary Methodology 1).

### Anthocyanin and Chlorophyll in Seedlings

Four replicates of 10 seeds per lot were sown in 500 mL polystyrene pots (8 pots per lot), filled with a mixture of pine bark, peat moss and vermiculite. Each pot contained 5 seeds. The seedlings were cultivated under controlled conditions of temperature (25°C), RH (50–70%) and white light (900 mm, LED lamps, 13 W) (Condado de Ilum., São Paulo, Brazil) with a photoperiod of 16/8 h light/dark. The pots were irrigated as needed. When the seedlings were well established, 7 days after sowing, the number of seedlings per pot was reduced to two, reducing overlap. Measurements were taken considering the canopy formed by the two seedlings in each pot, which totaled eight seedlings canopies per lot, taken 14 days after sowing.

The chlorophyll *a* index (Chl *a* index), anthocyanin index and the normalized difference vegetation index (NDVI) were calculated by a SeedReporter™ instrument (PhenoVation B.V., Wageningen, Netherlands). The Chl *a* index was estimated based on the reflectance at 710 and 770 nm (Gitelson et al., 2003), and the anthocyanin index from the reflectance at 540, 710, and 770 nm (Gitelson et al., 2009). The NDVI was calculated based on reflectance at 640 and 770 nm (Yengoh et al., 2015).

The initial fluorescence (F_0_), maximum fluorescence (F_m_), average chlorophyll *a* fluorescence and maximum quantum efficiency of photosystem II (F_v_/F_m_) were measured using a SeedReporter™ instrument, which is also integrated with high intensity amber LEDs (620 nm peak), with a saturating light intensity of 6.320 μmol m^–2^ s^–1^, while an interference filter (730 nm) transmitted the fluorescence signals from the leaves to a CCD chip. All parameters were calculated by SeedReporter™ software version 5.5.1.

### Further Experiments

This additional study was conducted with 300 seeds from one of the lots characterized as high quality (IAC OL3, lot 7) exposed to an artificial aging procedure (ISTA, 2020). The seeds were placed on a wire mesh suspended inside a covered plastic box containing 40 mL of distilled water at the bottom, providing a RH of 100%. Subsequently, the boxes were added to a B.O.D chamber set at 42°C. The seeds remained in these stress conditions for 24 and 48 h. A control group consisted of seeds not artificially aged. The objective was to induce seed deterioration by high temperature and high RH. Subsequently, the responses of the applied stress on pigment dynamics and seed brightness were investigated through multispectral images. To this end, stress-exposed and control seeds were subjected to evaluation of fluorescence chlorophyll *a*, fluorescence chlorophyll *b*, brightness (CIELAB L*) and anthocyanin index as described previously. These variables were also measured in seeds of another cultivar (IAC 503) exposed to the same stress conditions. Seeds belonging to the research lot and exposed to stress (IAC OL3; lot 7) were also used for seedling production following the same conditions previously described. At 14 days after sowing, chlorophyll *a* and anthocyanin indices, NDVI, F_0_, F_m_, chlorophyll *a* fluorescence and F_v_/F_m_ were calculated for each seedling using SeedReporter™ software.

### Statistical Design

The data obtained in the conventional tests performed for the seven seedlots were submitted to analysis of variance – ANOVA (*F* test; *p* ≤ 0.05) with four repetitions (*n* = 28). Comparison of means was performed by Tukey test (*p* ≤ 0.05). The data obtained from the multispectral images of 170 seeds of each lot were submitted to ANOVA and the Tukey test (each seed as a repetition; *n* = 170). The data obtained from multispectral images of the seedlings from the seeds of each lot were submitted to ANOVA and Tukey test with four replications (*n* = 28). The same analyses procedures were adopted for the data obtained in the further experiments. From the reflectance data (from 365 to 970 nm) observed for the seeds of each lot (*n* = 170), an interactive process analysis (*for loop*) was carried out in order to select the 20 combinations of 5 bands that best discriminated seedlots (660, 690, 780, 850, and 970 nm). The details of the computational procedures used are described in a supplementary file (Supplementary Methodology 2).

Principal component analysis (PCA) and correlation were performed with the data observed in conventional tests and multispectral images of the seeds. The Permanova test and the Bray-Curtis similarity index (Canoco 5 software) were used to identify the significance of the behavior observed in PCA between seedlots (*F* Test; *p* ≤ 0.05). Correlation analysis was calculated using the Spearman method, due to the non-normality of the variables. Additionally, when the variables were a different number of repetitions, the average was calculated, so a balanced observation could be made. The “ExpDes.pt” package of the R software was used to perform the analysis of variance (completely randomized design) and the Tukey test (R Core Team, 2021). The QDA analysis was performed with the MASS library (Venables and Ripley, 2002) with the MASS:qda() function, and the results of the confusion matrix and accuracy measurement were collected by the library and caret: confusionMatrix() (Kuhn, 2017).

## Results

### Physiological Quality and Physical Properties of Seeds

**FIGURE 1:**
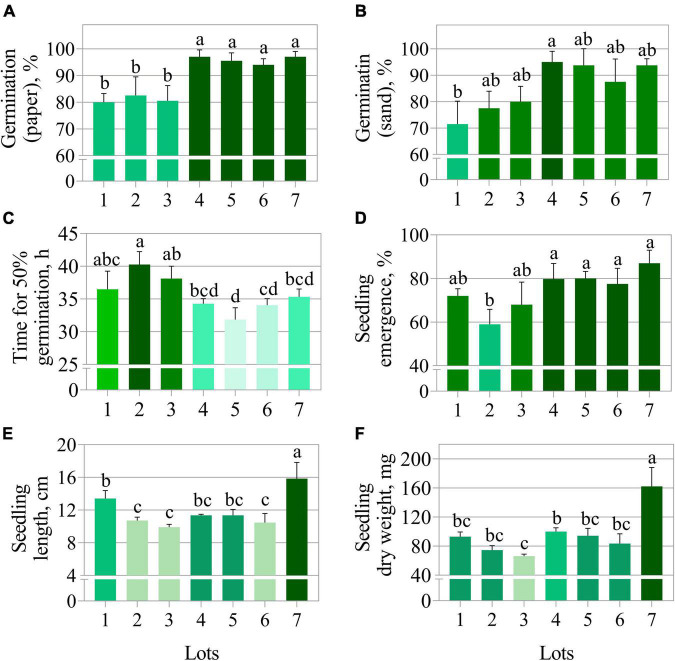
Physiological quality of seven seedlots of peanut (*Arachis hypogaea* L.; cv. IAC OL3) based on germination on paper **(A)**, germination on sand **(B)**, time for 50% germination **(C)**, seedling emergence **(D)**, seedling length **(E)**, and seedling dry weight **(F)**. Means (± standard deviation) with different letters indicate a significant difference (*p* ≤ 0.05).

**FIGURE 2:**
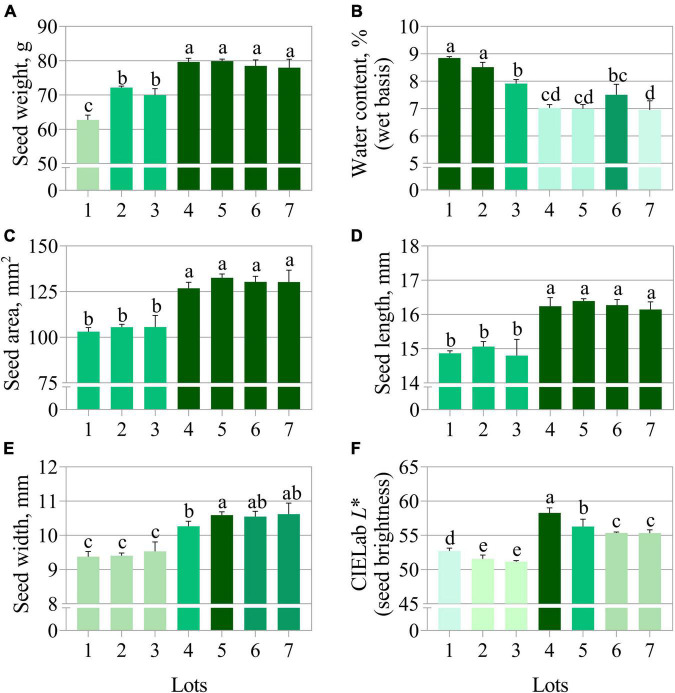
Physical properties of seven seedlots of peanut (*Arachis hypogaea* L.; cv. IAC OL3) based on fresh weight **(A)**, water content **(B)**, area **(C)**, length **(D)**, width **(E)**, and CIELab L* **(F)**. The CIELabL* represents the perceived brightness ranging from 0.0 (black) to 100.0 (white). Means (± standard deviation) with different letters indicate a significant difference (*p* ≤ 0.05).

The germination test using paper substrate clearly separated the seedlots into two groups, i.e., lots 1, 2, and 3 (lower quality) *vs.* lots 4, 5, 6, and 7 (higher quality) (Figure 1A). In contrast, the germination test using sand as substrate did not show a clear quality difference among seedlots (Figure 1B). The average time for 50% germination (t50) classified lot 2 as lower vigor (higher values of t50) (Figure 1C). In addition, seeds from lot 2 also presented the worst performance for seedling emergence and seedling length (Figures 1D,E). Nevertheless, lots 2 and 3 generated seedlings with very similar length as lot 6 (Figure 1E). The seedling length and dry weight measurements revealed lot 7 as having the best vigor (Figures 1E,F). Except for germination on paper (Figure 1A), conventional tests detected punctual and unclear differences in the quality of seedlots. Regarding the physical properties, seeds from lots 4, 5, 6, and 7 had higher fresh weight (Figure 2A) and this was associated with lower water content (≅ 7%) (Figure 2B). These seedlots in addition to the high quality indicated by the germination test (Figure 1A) also had superior area, length, width and brightness (CIELab L*) (Figures 2C–F).

### Seed Pigments

**FIGURE 3:**
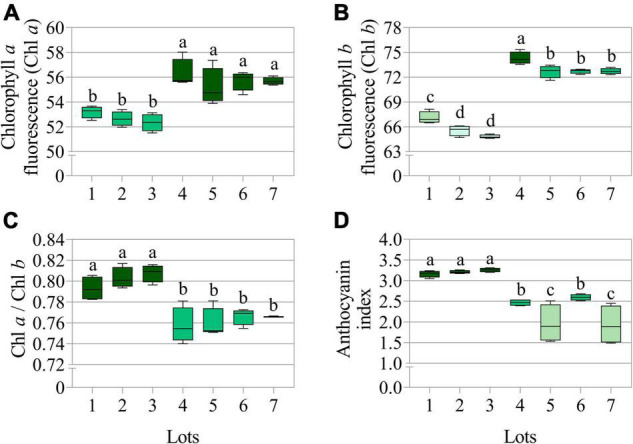
Average chlorophyll *a* fluorescence (Chl *a*) at 630/700 nm excitation/emission combination **(A)**, chlorophyll *b* fluorescence (Chl *b*) at 405/600 nm excitation/emission combination **(B)**, chlorophyll a/b ratio (Chl *a*/Chl *b*) **(C)**, and anthocyanin index **(D)** measured in seven seedlots of peanut (*Arachis hypogaea* L.; cv. IAC OL3). Means (± standard deviation) with different letters indicate a significant difference (*p* ≤ 0.05) (*n* = 170).

**FIGURE 4:**
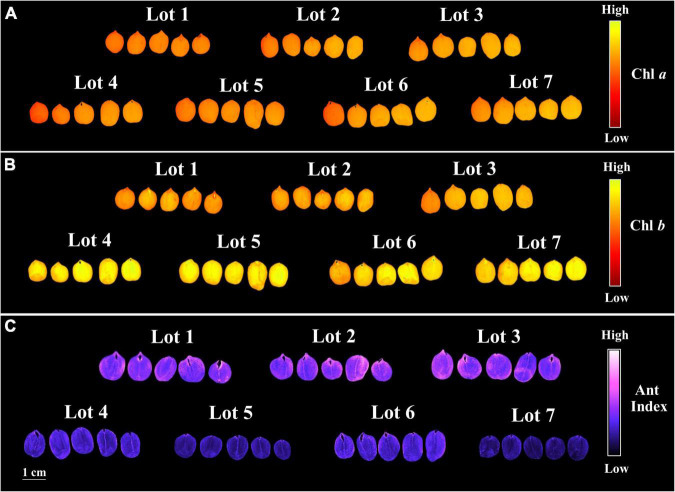
Chlorophyll a fluorescence (Chl *a*) at excitation/emission combination of 630/700 nm **(A)**, chlorophyll *b* fluorescence (Chl *b*) at excitation/emission combination of 405/600 nm **(B)**, and anthocyanin index (Ant Index) **(C)** of seven seedlots of peanut seeds (*Arachis hypogaea* L.; cv. IAC OL3). Each pixel in the images is represented by a unique value that corresponds to chlorophyll *a* and *b* fluorescence intensity or anthocyanin level.

The seedlots that exhibited the best performance in the germination test, i.e., lots 4, 5, 6, and 7 (Figure 1A) showed higher chlorophyll *a* and *b* fluorescence (Figures 3A,B), but a lower chlorophyll *a*/*b* ratio (Figure 3C) and anthocyanin index (Figure 3D). Therefore, the results indicated that there is a stronger difference in chlorophyll *b* between the two groups (lots 1, 2, 3 *vs.* lots 4, 5, 6, and 7), and this was also shown by comparing the chlorophyll *a* and *b* images (Figures 4A,B), in parallel with lower anthocyanins in the group with greater germination performance (Figure 4C).

**FIGURE 5:**
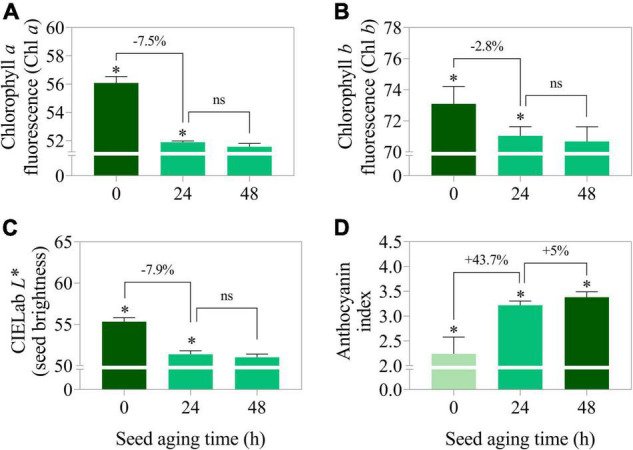
Chlorophyll *a* fluorescence at excitation/emission combination of 630/700 nm **(A)**, chlorophyll *b* fluorescence at excitation/emission combination of 405/600 nm **(B)**, CIELab L* representing the perceived brightness ranging from 0.0 (black) to 100.0 (white) **(C)**, and anthocyanin index **(D)** in peanut seeds (*Arachis hypogaea* L.; cv. IAC OL3) from lot 7 artificially aged for 0, 24, and 48 h. Means (± standard deviation); significant (*); not significant (ns); (p > 0.05) (*n* = 100).

**FIGURE 6:**
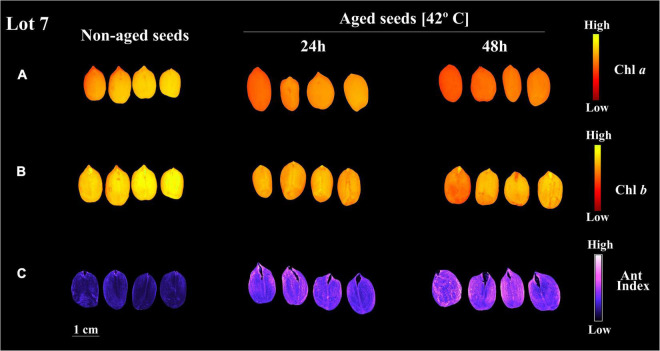
Chlorophyll *a* fluorescence (Chl *a*) at 630/700 nm excitation/emission combination **(A)**, chlorophyll *b* fluorescence (Chl *b*) at 405/600 nm excitation/emission combination **(B)**, and anthocyanin index (Ant Index) **(C)** in peanut seeds (*Arachis hypogaea* L.; cv. IAC OL3) from lot 7 for classes on non-aged seeds and seeds aged for 24 h and 48 h. Each pixel in the images is represented by a unique value that corresponds to chlorophyll *a* and *b* fluorescence intensity or anthocyanin level.

Curiously, when lot 7 was artificially aged, chlorophyll *a* and *b* fluorescence was rapidly reduced (Figures 5A,B, 6A,B). In addition, there was a reduction in the seed coat brightness (CIELab L*) (Figure 5C) and an increase in the anthocyanin index (Figures 5D, 6C). To verify whether this response can also occur in seeds of other genotypes, seeds obtained from IAC 503 cultivar were also artificially aged (Supplementary Figure 1). Likewise, there were lower chlorophyll *a* and *b* fluorescence signals, reduced seed coat brightness and increased anthocyanin index in aged seeds (Supplementary Figure 1).

### Seed Reflectance

**FIGURE 7:**
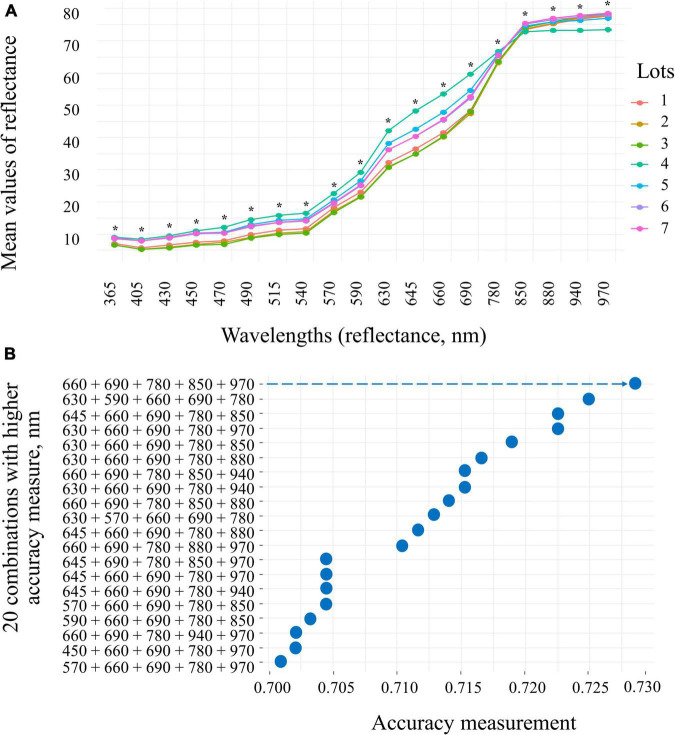
Reflectance spectral signature at 19 wavelengths (365 to 970 nm) of seven peanut seedlots (*Arachis hypogaea* L.; cv. IAC OL3) **(A)** and 20 combinations of wavelengths with distribution of accuracy determined by interactive process analysis **(B)**. The arrow indicates the combination of bands (660, 690, 780, 850, and 970) that showed the highest accuracy (0.730) for the subsequent analyses. *significant at the 0.05 probability levels (*n* = 170).

**FIGURE 8:**
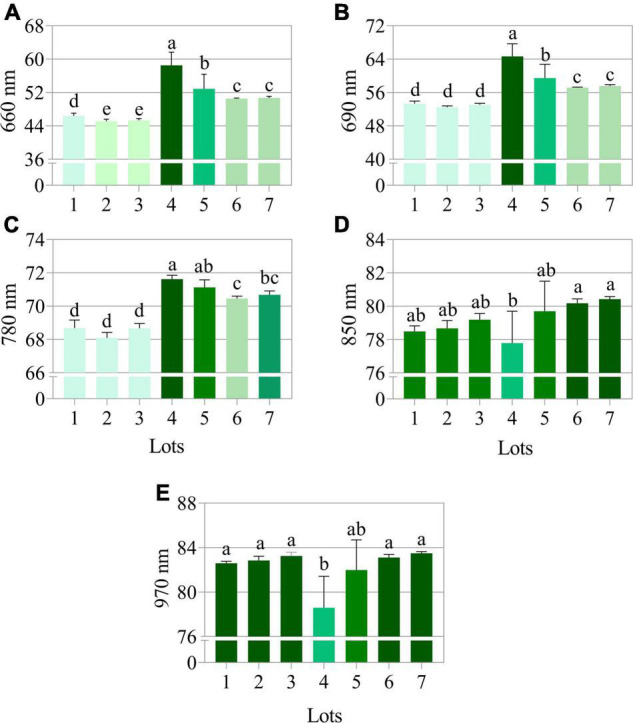
Reflectance mean of seven peanut seedlots (*Arachis hypogaea* L.; cv. IAC OL3) at **(A)** 660, **(B)** 690, **(C)** 780, **(D)** 850, and **(E)** 970 nm (previously shown as the best wavelengths to discriminate seeds as high and low vigor) (*n* = 170). Means (± standard deviation) with different letters indicate a significant difference (*p* ≤ 0.05).

The seeds with superior quality (lots 4, 5, 6, and 7) had the highest spectral signature in the visible region of the spectrum (405 to 540 nm; 630 to 780 nm) (Figure 7A). Seed reflectance was similar at longer wavelengths (850 and 970 nm), with the exception of lot 4 (Figure 7A). The combination of 660, 690, 780, 850, and 950 nm wavelengths showed superior accuracy to discriminate the spectral patterns of the seedlots (Figure 7B). When evaluating the bands individually, the results showed that the wavelengths of 660, 690, and 780 nm allow better separation of groups with lower and higher quality (lots 1, 2, and 3 *vs.* lots 4, 5, 6, and 7) (Figure 8).

### Correlation Between Physical, Physiological, Pigment and Reflectance Descriptors

**FIGURE 9:**
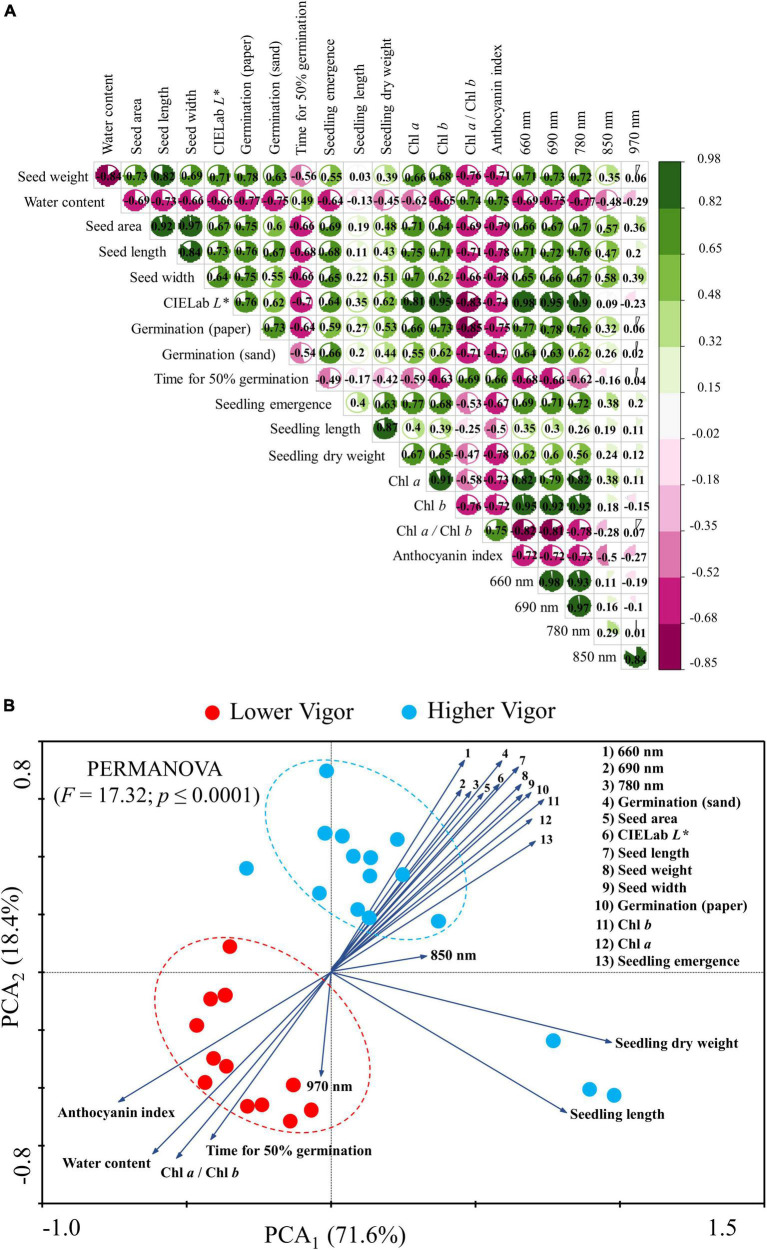
Correlation matrix **(A)** and biplots of principal component analysis (PCA) **(B)** for physical optical descriptors, physiological, pigment, and reflectance of peanut seeds (*Arachis hypogaea* L.; cv. IAC OL3) with lower (lots 1, 2, and 3; red circles) and higher vigor (lots 4, 5, 6, and 7; blue circles). The PCA vectors indicate the correlation between the classes (lower and higher vigor) and the dimensions PC_1_ and PC_2_. We used the PERMANOVA test and the Bray-Curtis similarity index in the PCA to identify the difference between seed classes at a 1% significance.

The correlation coefficients showed a relationship between physical descriptors and germination (paper): 0.78 (seed weight), –0.77 (water content) 0.75 (area), 0.76 (length), 0.75 (width) and 0.76 (CIELab L* – seed brightness). Seed brightness was the only physical descriptor with a correlation coefficient greater than 0.7 vs. t50 (vigor test). Between seed pigments and germination (paper) the correlations were: 0.73 (chlorophyll *b*), –0.85 (chlorophyll *a/*chlorophyll *b*), and –0.75 (anthocyanin index). The germination (paper) vs. reflectance bands obtained the following correlations: 0.77 (660 nm), 0.78 (690 nm), and 0.76 (780 nm). The correlation coefficients obtained for seedling emergence vs. 690 and 780 nm were 0.71 and 0.72, respectively. The reflectance bands showed the following correlations with seed brightness: 0.98 (660 nm), 0.95 (690 nm), and 0.9 (780 nm). The correlation between seed brightness and the seed pigments were: 0.81 (Chl *a*), 0.95 (Cha *b*), –0.83 (Chl *a*/Chl*b*), and –0.78 (anthocyanin index; Figure 9A).

The PCA allowed the correlation of the groups of seeds with high and low vigor (lots 1, 2, 3 vs. lots 4, 5, 6, and 7), explaining 71.6% of the significant variation (PCA_1_) found (PERMANOVA; *p* < 0.001). Most of the seeds with lower vigor were negatively correlated with the anthocyanin index, water content, chlorophyll *a*/*b* ratio, time for 50% germination and reflectance at 970 nm. Meanwhile, the group of seeds with higher vigor exhibited positive correlation with all other variables as seed weight, area, length, width, brightness (CIELab L*), chlorophyll *a*, chlorophyll *b*, and seed reflectance (660, 690, and 780 nm). These variables were expressed to a higher degree (vector modulus) in high vigor seedlots (lots 4, 5, 6, and 7) jointly with germination in paper, germination in sand, and seedling emergence (Figure 9B).

### Seed Quality Classification Based on Machine Learning Models Using Physical Properties, Pigments and Reflectance Descriptors

From the seed groups (Figure 9B) divided into high vigor (lots 1, 2, and 3) and low vigor (lots 4, 5, 6, and 7) quadratic discriminant analysis (QDA) models were constructed. Based on the data set (*n* = 1190), the first model generated using the physical optical descriptors (area, length, width, and CIELab L*) was able to predict the behavior of the two seed groups (high and low vigor) with 89% accuracy. For the second model, using seed pigments (chlorophyll *a*, chlorophyll *b*, and the anthocyanin index), the accuracy was 94%. Using the most significant wavelengths of reflectance (660, 690, 780, 850, and 970 nm) the accuracy was 97%. From the union of the physical optical descriptors, pigments and reflectance of the seeds in a single model, the accuracy was 98% (Table 1).

**TABLE 1:** Quadratic discriminant analysis (QDA) based on physical optical descriptors, pigments and reflectance of peanut seedlots (*Arachis hypogaea* L.; cv. IAC OL3) for groups of lower and higher vigor.

Predictor variable: area, length, width and CIELab L* (physical optical descriptors)						
Seedlot groups*	**Training set (*n* = 833)^1^**			**Validation set (*n* = 357)^1^**		
	**Lower Vigor**	**Higher Vigor**	**Accuracy**	**Lower Vigor**	**Higher Vigor**	**Accuracy**
Lower Vigor	0.94	0.14	0.91	0.93	0.15	0.89
Higher Vigor	0.06	0.86		0.07	0.85	

**Predictor variable: Chlorophyll *a*, Chlorophyll *b* and anthocyanins (pigments)**						

Seedlot groups*	**Training set (*n* = 833)^1^**			**Validation set (*n* = 357)^1^**		
	**Lower Vigor**	**Higher Vigor**	**Accuracy**	**Lower Vigor**	**Higher Vigor**	**Accuracy**

Lower Vigor	0.91	0.06	0.93	0.96	0.07	0.94
Higher Vigor	0.09	0.94		0.04	0.93	

**Predictor variable: 660, 690, 780, 850, and 970 nm (reflectance)**						

Seedlot groups*	**Training set (*n* = 833) ^1^**			**Validation set (*n* = 357)^1^**		
	**Lower Vigor**	**Higher Vigor**	**Accuracy**	**Lower Vigor**	**Higher Vigor**	**Accuracy**

Lower Vigor	0.98	0.04	0.97	0.99	0.05	0.97
Higher Vigor	0.02	0.96		0.01	0.95	

**Predictor variable: physical optical descriptors, pigments and reflectance**						

**Seedlot groups**	**Training set (*n* = 833) ^1^**			**Validation set (*n* = 357) ^1^**		
	**Lower Vigor**	**Higher Vigor**	**Accuracy**	**Lower Vigor**	**Higher Vigor**	**Accuracy**

Lower Vigor	0.99	0	0.99	0.98	0.02	0.98
Higher Vigor	0.01	1		0.02	0.98	

*Lower Vigor: lots 1, 2, and 3; Higher Vigor: lots 4, 5, 6, and 7.

*^1^From the dataset observed in all seedlots (n = 1190), 70% (n = 833) were randomly sampled for training assessment and 30% for validation (n = 357).*

### Pigments and Photosynthetic Efficiency of Seedlings

**FIGURE 10:**
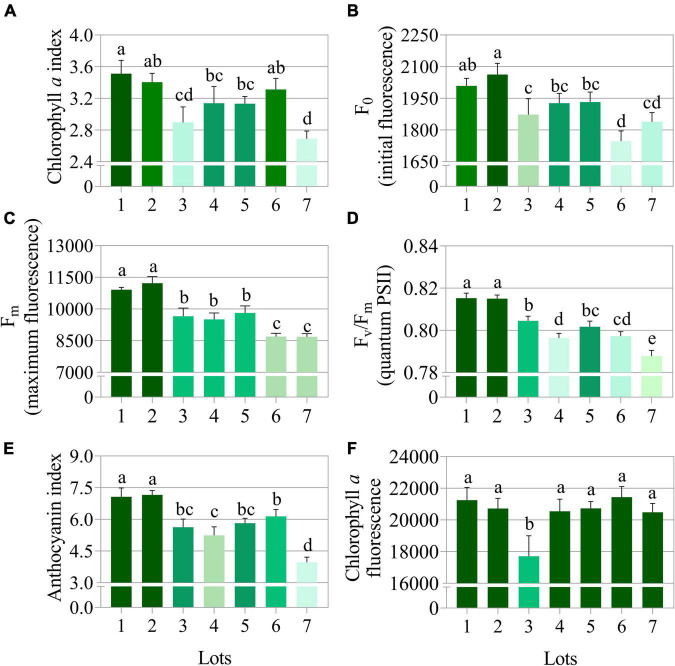
Photosynthetic activity measured by chlorophyll *a* index **(A)**, initial fluorescence (F_0_) **(B)**, maximum fluorescence (F_m_) **(C)**, quantum yield of photosystem II measured by F_v_/F_m_ **(D)**, anthocyanin index **(E)**, and chlorophyll *a* fluorescence **(F)** in peanut seedlings (*Arachis hypogaea* L.; cv. IAC OL3) at 14 days after sowing: excitation of chlorophyll molecules were induced at 620 nm and emission at 700 nm. Means (± standard deviation) with different letters indicate a significant difference (p ≤ 0.05). Peanut seedlings were obtained from seeds of lower (Lots 1, 2, and 3) and higher vigor (Lots 4, 5, 6, and 7).

**FIGURE 11:**
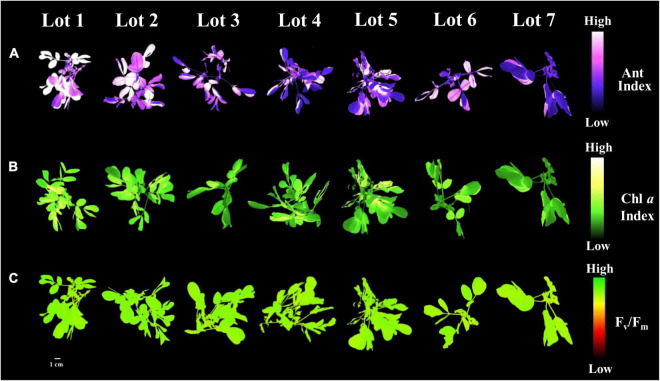
Anthocyanin index **(A)**, chlorophyll *a* index **(B)**, and maximum quantum efficiency of photosystem II based on F_v_/F_m_ **(C)** in peanut seedlings (*Arachis hypogaea* L.; cv. IAC OL3) from seedlot 7. The pigments and photosynthetic efficiency of peanut seedlings were evaluated at 14 days after sowing of the seeds from the seven lots studied. Each pixel in the image is represented by a unique value that corresponds to fluorescence intensity; higher pixel values indicate higher anthocyanin, fluorescence and F_v_/F_m_ intensity.

The low vigor seedlots (i.e., Lot 1) generated seedlings with higher values for the variables chlorophyll *a* index, initial fluorescence and maximum fluorescence, and F_v_/F_m_ ratio (Figures 10A–D). Seedlings from these seeds also had a high anthocyanin index (Figure 10E). Chlorophyll *a* fluorescence was similar among most of the seedlings from the analyzed seedlots (Figure 10F). Differences in the anthocyanin index and the chlorophyll *a* index of the seedlings were most evident between the high and low vigor seedlots 1 and 7 (Figures 11A,B). The F_v_/F_m_ ratio was very precise to show differences in photosynthetic activity by images of the evaluated seedlings (Figure 11C).

**FIGURE 12:**
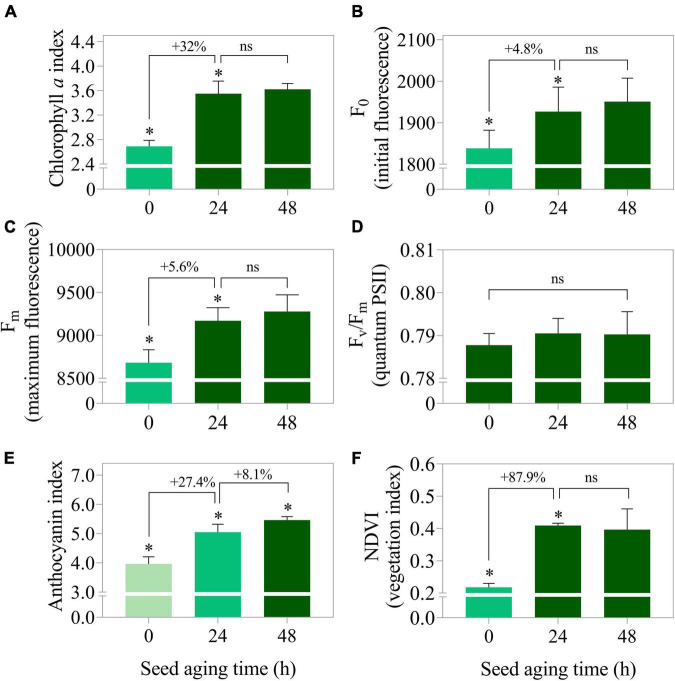
Photosynthetic activity measured by chlorophyll *a* index **(A)**, initial fluorescence (F_0_) **(B)**, maximum fluorescence (F_m_) **(C)**, photosystem II quantum yield measured by F_v_/F_m_ **(D)** as well as stress indicators such as anthocyanin index **(E)**, and normalized vegetation index (NDVI) **(F)** in peanut seedlings (*Arachis hypogaea* L.; cv. IAC OL3) at 14 days after sowing: excitation of chlorophyll molecules were induced at 620 nm and emission at 700 nm. Means (± standard deviation). Asterisks (*) indicate significant differences (*p* ≤ 0.05). Peanut seedlings were obtained from lot 7 seeds after aging times (0 h, 24 h, and 48 h at 42°C).

**FIGURE 13:**
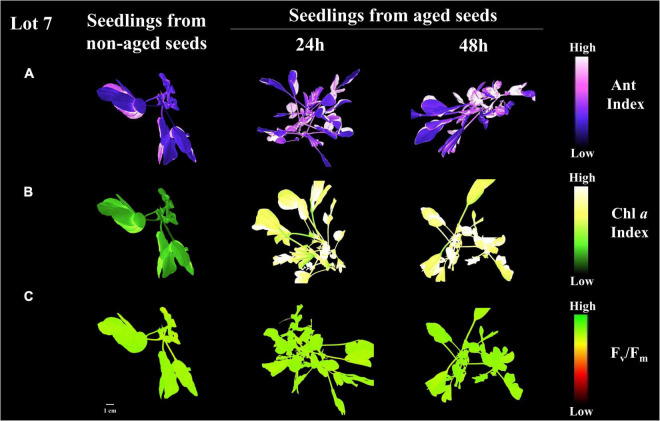
Anthocyanin index **(A)**, chlorophyll α index **(B)**, and maximum quantum efficiency of photosystem II based on F_v_/F_m_ **(C)** in peanut seedlings (*Arachis hypogaea* L.; cv. IAC OL3) from seeds of lot 7 after artificial aging (24 h and 48 h at 42°C). Each pixel in the image is represented by a unique value that corresponds to fluorescence intensity; higher pixel values indicate higher anthocyanin, fluorescence and F_v_/F_m_ intensity.

Seedlings from seedlot 7 that were submitted to artificial aging showed improvement in the main photosynthetic parameters. The chlorophyll *a* index, initial fluorescence and maximum fluorescence increased by 32, 4.8 and 5.6% after 24 h of stress, respectively (Figures 12A–C). The time of seed exposure to aging did not affect the quantum yield of the photosystem II system (F_v_/F_m_) of the seedlings (Figure 12D). However, it caused an increase in the anthocyanin index and the normalized vegetation index (Figures 12E,F). This behavior was clearly reflected in the images (Figures 13A–C).

## Discussion

This study contains contributions that highlight the accuracy of technologies based on multispectral images and machine learning to identify peanut seeds with superior quality. New evidence reinforces the possibility of autonomous detection of physical parameters, chlorophyll fluorescence and light reflectance in peanut seeds to assess their physiological quality. Here, these and other original data address the use of post-harvest technologies to advance the peanut seed production sector in the world.

### Seed Quality

The seed industry performs the physiological quality control of lots every cultivation season. Among the conventional tests capable of assessing seed quality, germination performed within 10 days provides sufficiently satisfactory results (Figure 1A). In the case of t50 (vigor test), the distinction of seedlots with high and low vigor is also possible (Figure 1B). However, these are tests that require a lot of time and effort to be performed on a large scale. This makes the process of seed quality control inefficient. Regarding water content, the low moisture observed in certain lots (Figure 2A) is described as a state that slows the natural deterioration processes (Buitink and Leprince, 2004) in addition to prolonging the conservation of seeds in storage (Leprince et al., 2017). Therefore, evaluation is essential for obtaining seedlots with high quality. Still, it is a destructive methodology and, as with the other conventional tests, depends on human analytical ability. With this in mind, based on studies with seeds of other species (Mortensen et al., 2021) the potential of multispectral images technologies was investigated. New markers capable of efficiently determining peanut seed quality were found.

A first component of this approach comprises physical properties (shape and brightness). Characteristics such as area, length and width have been positively associated with seed vigor and adequate seedling establishment. In fact, peanut seeds with high quality showed additional dimensions (Figures 2C–E), and that possibly gave them a higher proportion of reserves to subsidize germination, such as lipids (Zhou et al., 2019). It has also been found that lower exposure of soybean seeds to stress situations, such as radiation (Oliveira et al., 2021), preserves their brightness characteristics. In alfalfa, it has been shown that the natural aging of seeds itself interferes with this aspect (Wang X. et al., 2021). It is worth noting that the reduction in tegument brightness is a common phenomenon in other species, such as beans (Piotrowicz-Cieślak et al., 2020), and may indicate the advancement of oxidative processes associated with seed deterioration (Erfatpour et al., 2021). In orthodox seeds, such as peanuts, seed deterioration occurs in progressive stages at the cellular level and results in loss of vigor (Ebone et al., 2019). Thus, the physical variables explored in this work through multispectral images demonstrated potential for quality control during the processing of peanut seedlots.

### Seed Pigments

In addition to the above physical properties, pigments in peanut seeds have also been found to add useful information for the seed industry. In an initial explanation, it can be pointed out that high quality seedlots may contain extra volume of both reserves and pigments (Figures 3A,B) due to their higher weight and area (Figures 2B,C). In fact, the low relation between chlorophyll *a/b* (Figure 3C) indicated a higher proportion of chlorophyll *b* in high quality lots (Figure 4B). In senescent plant tissues, the reduction in chlorophyll fluorescence is described as a deteriorating process (Donaldson and Williams, 2018; Donaldson, 2020). From this perspective, peanut seed quality may be directly associated with chlorophyll fluorescence dynamics. It may also be associated to the accumulation of anthocyanins (Figures 3D, 4B,C) since the biosynthesis of this flavonoid is part of the secondary metabolism of plants against stress (Liu et al., 2018). Further studies were conducted in order to understand whether pigment dynamics in peanut seeds interfere with their quality. For this purpose, seeds from one of the lots identified as high quality (high germination and vigor) were exposed to controlled stress (artificial aging).

Stress applied to peanut seeds (aged seeds) caused changes in pigment dynamics (chlorophylls fluorescence and anthocyanin index) and brightness (CIELab L*). Considering mature, non-greenish soybean seeds, the fluorescence of chlorophylls (residual in the embryo) decreases as the artificial aging process under high temperature and high RH progresses (Barboza da Silva et al., 2021b). Furthermore, the increased exposure of seeds to this stress (high temperature and high RH) reduces their ability to form vigorous seedlings. It has been demonstrated that mature soybean seeds with reduced germination have lower chlorophyll fluorescence characteristics than seeds with higher viability (Li et al., 2019). Thus, the possibility exists that the loss of fluorescence occurs as seeds age. In plants, this has been documented for leaf tissues in advanced senescence (Donaldson, 2020). In this work, the reduction in chlorophyll fluorescence and brightness of seeds exposed to stress (IAC OL3 and IAC 503) reinforces the idea that the degree of deterioration or aging of peanut seeds alters their spectral properties. Taking these observations into consideration, pigment dynamics and seed brightness can be indicators of seed quality. Also, both reveal the degree of stress accumulated in seed tissues. In the peanut seed industry, technologies that detect these characteristics through multispectral images have a promising potential to improve lot quality control and making it more accurate.

### Seed Reflectance

Another promising possibility for assessing seed quality was found in this work through reflectance. Higher quality lots were formed by seeds with high reflectance at wavelengths between 660 and 780 nm (Figures 8A,B). The peculiarities of seeds, such as chemical composition, color and other attributes, are known to interfere in the absorbance and reflectance dynamics of incident light (Elmasry et al., 2019a). It is worth noting that high quality peanut seeds contained a naturally enhanced chlorophyll fluorescence, especially Chl *b*, and higher tegument brightness (Figure 2F). In this context, these characteristics can have contributed to the increased light reflected by the better-quality seeds, thus defining their high reflectance pattern in specific bands (Figures 8A–C). Apparently, this behavior is not a common and interspecific rule in nature. As an example, *Jatropha curcas* seeds have superior quality associated with enhancement in their lipid content, which results in low reflectance in the near infrared range (940 nm) (Bianchini et al., 2021). In tomato seeds, on the other hand, this high performance and low reflectance are linked to embryo maturity and protective pigments that absorb more light in the UV spectrum (365 nm) (Galletti et al., 2020). Here, the physiological quality attributes (germination and vigor) were associated with high reflectance at specific wavelengths (660 to 780 nm), so far not considered for peanut seeds. It is worth noting that in the plant domain (bermudagrass), higher reflectance values (900/970 nm) can be strongly associated with leaf water content under water stress conditions (Caturegli et al., 2020). Therefore, the reflectance patterns obtained in this work show a singular behavior with a unique competence to define the physiological quality of peanut seeds.

### Data Correlation and Seed Quality Classification Using Machine Learning

Summarizing our findings, it is worth highlighting the significant correlations between physical optical parameters (area, length, width and brightness – CIELab L*), pigments (chlorophyll fluorescence and anthocyanin) and reflectances (660, 690, and 780 nm) with germination and seed vigor (Figure 10A). These results establish an unprecedented connection between tests performed to assess seed quality with multispectral images parameters, with the goal of categorizing seedlots with high quality. Furthermore, they demonstrated the robustness of potential markers of peanut seed physiological quality found through non-invasive technologies. The principal component analysis method proved to be an efficient technique for interpreting the behavior of seedlots (high and low vigor). The gain in the ability to manage datasets using PCA has been highlighted (Taheri-Garavand et al., 2021c). However, it should be considered that the manual management of the volume of data generated through multispectral seed images can hinder decision making in routine analyses in the seed industry. Separating the behavior of the seedlots into groups of low and high vigor (Figure 10B) brought up the following question: in practice, how can these differences in seed quality be quickly diagnosed using only the generated database containing all multispectral image parameters found? With this in mind, ways to automatically recognize seeds of high and low quality were tested using computational resources of high predictive accuracy.

From the multispectral seed dataset, the surprising sensitivity of machine learning algorithms based on the QDA method (Table 1) was verified for autonomous recognition of patterns identified in conventional seed quality analysis (Figure 10B). It is worth noting that the QDA method is quite robust to data non-normality (lower error probability), except when distributions are highly asymmetric (Clarke et al., 1979), different from what was observed here (Supplementary Methodology 1). Also, it is an efficient parametric method because it takes into account the low variability when different data sets are used to build prediction models (James et al., 2021). The QDA method has been used successfully in the field of Plant Science, with examples ranging from protein structure classification (Yuan et al., 2017) to phytosanitary diagnosis from plant oil dielectric properties (Khaled et al., 2018). The use of the QDA method as part of an artificial intelligence strategy applied to post-harvest proved to be a powerful tool for categorizing the quality of peanut seedlots.

This possibility of automation was successfully explored in previous studies for the analysis of image parameters of seeds from other crops (Elmasry et al., 2019a; Mortensen et al., 2021). In species such as soybean (Baek et al., 2019; Medeiros et al., 2020a), cowpea (Rego et al., 2020), oat (França-Silva et al., 2020), *U. brizantha* (Medeiros et al., 2020b) and corn (Wang Z. et al., 2021), the ability of algorithms to detect spectral features of seeds with high accuracy (above 90%) through images was proven. Taking this knowledge into consideration in addition to the findings of this work (Table 1), it is clear that part of the modernization process of the seed production sector in the world can be based on the use of multispectral image technologies. In the peanut production chain, these devices capable of capturing images in the UV, visible and near-infrared range have the potential to promote strategies to mitigate the incidence of seeds with low vigor in commercial lots. This problem, in addition to hampering the proper formation of a crop (Carter et al., 2019), can lead to a higher number of seeds needed to meet the intended plant stand. At this point, artificial intelligence resources have shown to be highly capable of improving seed quality management programs based on detailed and real-time diagnosis of the seedlots.

### Pigments and Photosynthetic Efficiency of Seedlings

In face of the primary technological aim of the seeds, which is the establishment of a seedling, its association with seed quality was investigated. Interestingly, seeds of low physiological quality gave rise to seedlings with superior photosynthesis parameters (Figures 11A–D). Even with the enhancement of the photosynthetic potential, there was an increase in the anthocyanin index in the leaves (Figure 11E), which indicates some degree of stress (Liu et al., 2018). The outcome of these results motivated us to think about whether there is an intrinsic protection mechanism in peanut seeds that helps the establishment of the seedling with low vigor. This can be a natural survival strategy in unfavorable situations (stress), which optimizes the chances of perpetuating the species in the cultivation environment, as discussed for other species (Marcos et al., 2018a,b). A similar proposal was explored in tomato (Nogueira et al., 2021), and it was found that seeds produced in a stressful environment gave rise to seedlings with adaptive enhancement in chlorophyll fluorescence. Thus, considering the notable connection of chlorophyll fluorescence found (Figure 11A) with photosynthesis in plant organisms (Valcke, 2021), the idea that peanut seedlings signaled compensatory adjustments in photosynthetic capacity in response to seed deterioration induced by artificial aging (high temperature and high RH) was proposed. In order to better understand these concepts, seedlings from seeds exposed to stress (24 and 48 h at 42°C/100% RH) were produced and assessed for their photosynthetic capacity as well as stress indicators (anthocyanin and normalized vegetation indices).

Surprisingly, after 24 h of artificial aging of high-quality seeds (lot 7), there was a proportional enhancement in the photosynthetic parameters of the seedlings (Figures 12A–C), besides an evident increase in leaf stress indices (Figures 12D,E). It is interesting to think that if the deteriorated seeds were really conditioned to access stress repair mechanisms, in practice the low quality of seedlots would naturally be compensated without harming the establishment of seedlings. On the other hand, seeds in this condition can lead to failures in the stand due to a higher incidence of abnormal seedlings and/or non-viable seeds (Supplementary Figure 2). Thus, seeds of low vigor should not be used for the installation of tillage, since the negative reflexes of the failures they cause in the plant stand extend to the harvest and reduce grain yield (Bagateli et al., 2019; Ebone et al., 2020). In this way, multispectral images of seedlings can provide information associated with their photosynthetic apparatus with the reverse logic of what happens in seeds (Figure 13B). For this reason, they need prior knowledge of the level of seed deterioration to effectively contribute as a marker of the physiological quality of seedlots. Still, stress indicators such as the levels of anthocyanins found (Figures 12D, 13A) connected more directly with what occurs in seeds (Figure 5D). Such results have the potential to anticipate the behavior of post-germination events and integrate robust quality control programs associated with seedling establishment. Also, allows the prediction of physiological dysfunctions associated with seed deterioration and the initial photosynthetic behavior of a crop in the field, which deserves to be explored in future investigations.

### Perspectives

These are innovative techniques to assess the quality of peanut seedlots in a non-destructive and accurate way. The possibility of providing farmers with seeds that are highly capable of generating productive plants makes the search for these innovations one of the technological priorities in agriculture. Multispectral images represent a sensory bridge that extends human vision to access information hitherto unexplored in peanut seeds. A practical example is that through images, seedlots of lower quality can be identified. They generate seedlings with higher levels of stress (anthocyanins). Therefore, these lots can be allocated to less stressful cultivation environments in order to take advantage of the seed stock, within a certain quality level, and mitigate possible losses in the future crop. From the quality markers found, improvement solutions can be thought along the peanut production chain, from classification in processing to seed quality control. There is also, the opportunity to carry out these steps autonomously through machine learning models (QDA method). On a commercial scale, a capital investment is initially required to adopt the approach employed (Taheri-Garavand et al., 2021a). However, the wide applications of these technologies in the seed industry can bring significant returns through two aspects: (i) increased efficiency of post-harvest processes and, consequently, (ii) cost reduction.

## Conclusion

New markers that effectively track peanut seed quality were found. The combination of physical properties (area, length, width, and coat brightness), pigments (chlorophyll fluorescence and anthocyanin), and light reflectance (660, 690, and 780 nm), is highly efficient to identify peanut seedlots with superior quality (98% accuracy). Regarding seedlings, stress indicators such as anthocyanins directly reflect the quality of the seedlots. The association of these markers with artificial intelligence highlights the potential for automation of post-harvest processes integrated with quality analysis logistics in the peanut seed industry. Overall, our findings provide valuable insights for managing the quality attributes of one of the most essential inputs to the world’s agricultural activity: the seed.

## Data Availability Statement

The original contributions presented in the study are included in the article/Supplementary Material, further inquiries can be directed to the corresponding author.

## Author Contributions

GF, CM, and EA generated the research ideas. GF collected seed physiological quality data and wrote and formatted the manuscript. CM and JS collected multispectral image analysis data. WH and GF analyzed the data. CM, TB, AP, CC, and EA reviewed the manuscript, rewriting, discussing, and commenting. All authors read and approved the final manuscript.

## Conflict of Interest

The authors declare that the research was conducted in the absence of any commercial or financial relationships that could be construed as a potential conflict of interest.

## Publisher’s Note

All claims expressed in this article are solely those of the authors and do not necessarily represent those of their affiliated organizations, or those of the publisher, the editors and the reviewers. Any product that may be evaluated in this article, or claim that may be made by its manufacturer, is not guaranteed or endorsed by the publisher.

## Footnotes

1
https://copercana.com.br

2
http://www.coplana.com
