# Supplementary material for: An Approach Using Emerging Optical Technologies and Artificial Intelligence Brings New Markers to Evaluate Peanut Seed Quality
Source: Front Plant Sci. 2022 Apr 14;13:849986. doi: 10.3389/fpls.2022.849986 (PMC9048030; doi:10.3389/fpls.2022.849986)
Supplement: Supplementary file 1 [file Table_1.DOCX]

**Supplementary Table 1.** Details regarding the variables measured, number of seeds, and method used to evaluate peanut seeds and seedlings.

| Variables | Number of seeds | Method |
| --- | --- | --- |
| **Conventional tests - seeds** | | |
| Water content | 40 | ISTA (2020) |
| Germination (paper) | 100 |  |
| Germination (sand) | 100 |  |
| Time for 50% germination | 100 | Joosen et al (2010) |
| Seed weight | 100 | Krzyzanowski et al (2020) |
| Seedling emergence | 100 |  |
| Seedling length | 40 |  |
| Seedling dry weight | 40 |  |
| **Multispectral images - seeds** | | |
| Seed area | 170 | * |
| Seed length | 170 |  |
| Seed width | 170 |  |
| CIELab *L** (Seed brightness) | 170 | Oliveira et al (2021) |
| Chlorophyll fluorescence *a* | 170 | Barboza da Silva et al (2021) |
| Chlorophyll fluorescence *b* | 170 |  |
| Chl a / Chl b | 170 | * |
| Anthocyanin index | 170 | Galletti et al (2020) |
| Reflectance | 170 |  |
| **Multispectral images - seedlings** | | |
| Chlorophyll *a* index | 40 | Galletti et al (2020)  Oliveira et al (2021) |
| F0 | 40 |  |
| Fm | 40 |  |
| Fv/Fm | 40 |  |
| Anthocyanin index | 40 |  |
| Chlorophyll fluorescence *a* | 40 |  |

* Variables obtained from the methodology presented in this paper.
