# Supplementary material for: An Approach Using Emerging Optical Technologies and Artificial Intelligence Brings New Markers to Evaluate Peanut Seed Quality
Source: Front Plant Sci. 2022 Apr 14;13:849986. doi: 10.3389/fpls.2022.849986 (PMC9048030; doi:10.3389/fpls.2022.849986)
Supplement: Supplementary file 2 [file Table_2.DOCX]

**Supplementary Table 2.** Further experiments: details regarding the variables measured, number of seeds, and method used for each multispectral test with peanut seeds and seedlings.

| Variables ^1^ | Number of seeds | Method |
| --- | --- | --- |
| **Seeds** | | |
| CIELab *L** (Seed brightness) | 300 | Oliveira et al (2021) |
| Chlorophyll fluorescence *a* | 300 | Barboza da Silva et al (2021) |
| Chlorophyll fluorescence *b* | 300 |  |
| Anthocyanin index | 300 | Galletti et al (2020) |
| **Seedlings** | | |
| Chlorophyll *a* index | 40 | Galletti et al (2020)  Oliveira et al (2021) |
| F0 | 40 |  |
| Fm | 40 |  |
| Fv/Fm | 40 |  |
| Anthocyanin index | 40 |  |
| Chlorophyll fluorescence *a* | 40 |  |

1 These variables were measured after artificial aging of the seeds for 24h and 48h under high temperature (42ºC) and high relative humidity (100%). The seeds came from lot 7 of the research, previously characterized with high germination and vigor.
