# Supplementary material for: An Approach Using Emerging Optical Technologies and Artificial Intelligence Brings New Markers to Evaluate Peanut Seed Quality
Source: Front Plant Sci. 2022 Apr 14;13:849986. doi: 10.3389/fpls.2022.849986 (PMC9048030; doi:10.3389/fpls.2022.849986)
Supplement: Supplementary file 3 [file Data_Sheet_1.docx]

**Supplementary methodology 1**

Machine learning models (Quadratic Discriminant Analysis - QDA) were calculated for each group of seedlots (high and low vigor) using the parameter *quadratic discrimination score* (Hastie et al., 2009) as expressed below.

δ_k_(x) **=** - $\frac{1}{2}$ log | $\sum_{k}$| - $\frac{1}{2}$ $(x- \mu_{k})$ ^⊤^ $\sum_{k}^{-1} \left( x- \mu_{k} \right)$ + log$\frac{N_{k}}{N}$ (1)

where:

δ_k_(x): decision threshold parameters;

∑_k_: Covariance matrix of the k-th group;

x: vector of observed values;

μ_k_: vector of means of the k-th group;

N_k_: number of observations belonging to the k-th group;

The classes used were k = 1, 2 (low vigor and high vigor), and using QDA over LDA was chosen due to the inhomogeneous variance and the covariance matrix structure, according to the following expression:

$\sum_{k}\neq\sum$ for ∀_k_ (2)

Thus, if the observation belongs to the first group, the classification rule is given by:

δ_1_(x) − δ_2_(x) > 0 (3)

Some groups of variables did not present the multivariate normal distribution in the parametric probability space threshold. Still, QDA was chosen due to its parametric method, when compared to other non-parametric methods, because it takes into account the low variability when different data sets are used. In addition, according to Clarke et al. (1979) non-normality ends up being more affected in cases where the marginal distribution has asymmetric behavior. This is contrary to what was observed in this paper, as presented below (Figure 1, 2 and 3).


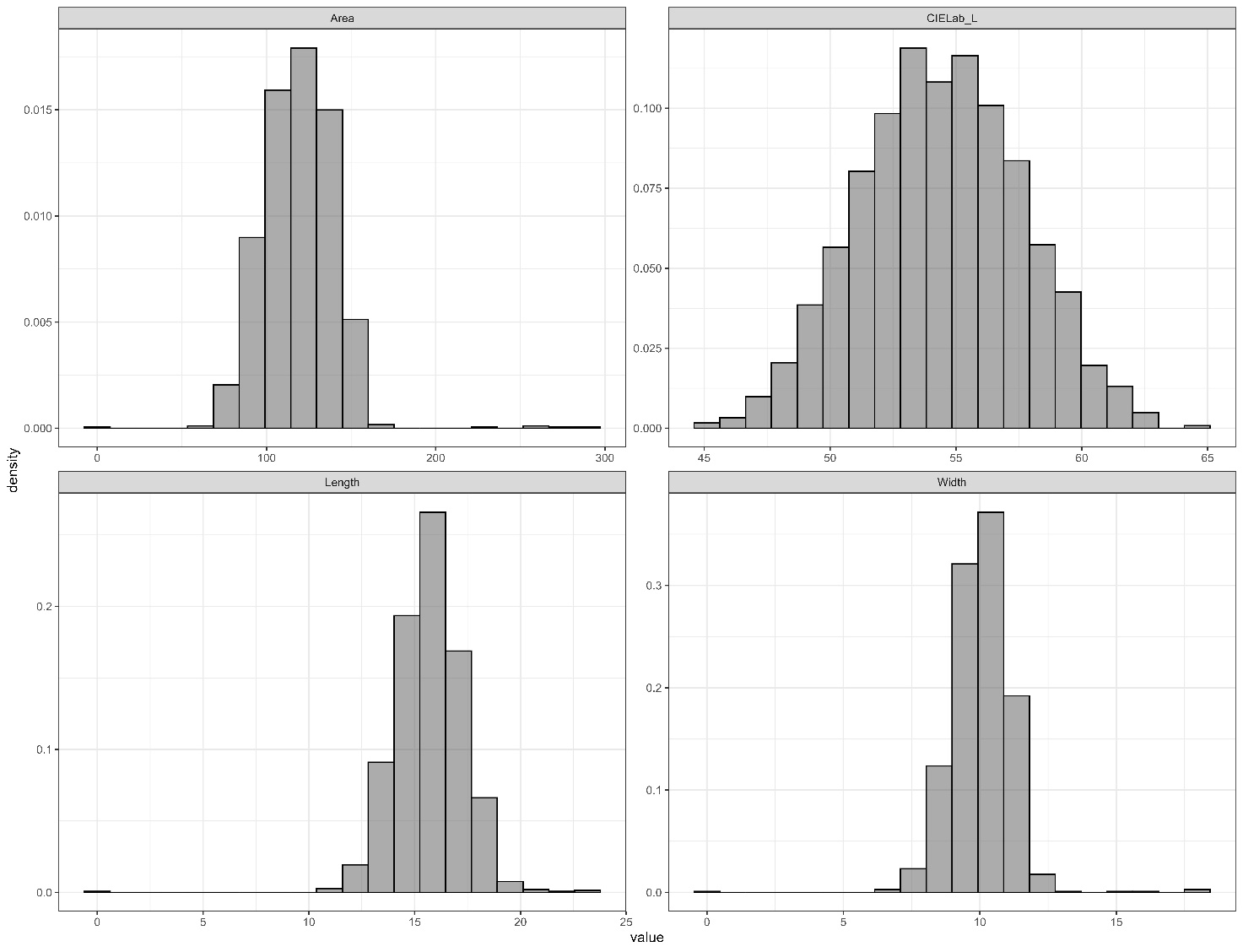


**Figure 1**. Density curve for the predictor variables Area, CIELab *L**, Length and Width of peanut seeds that were used for the machine learning models obtained with the QDA method.


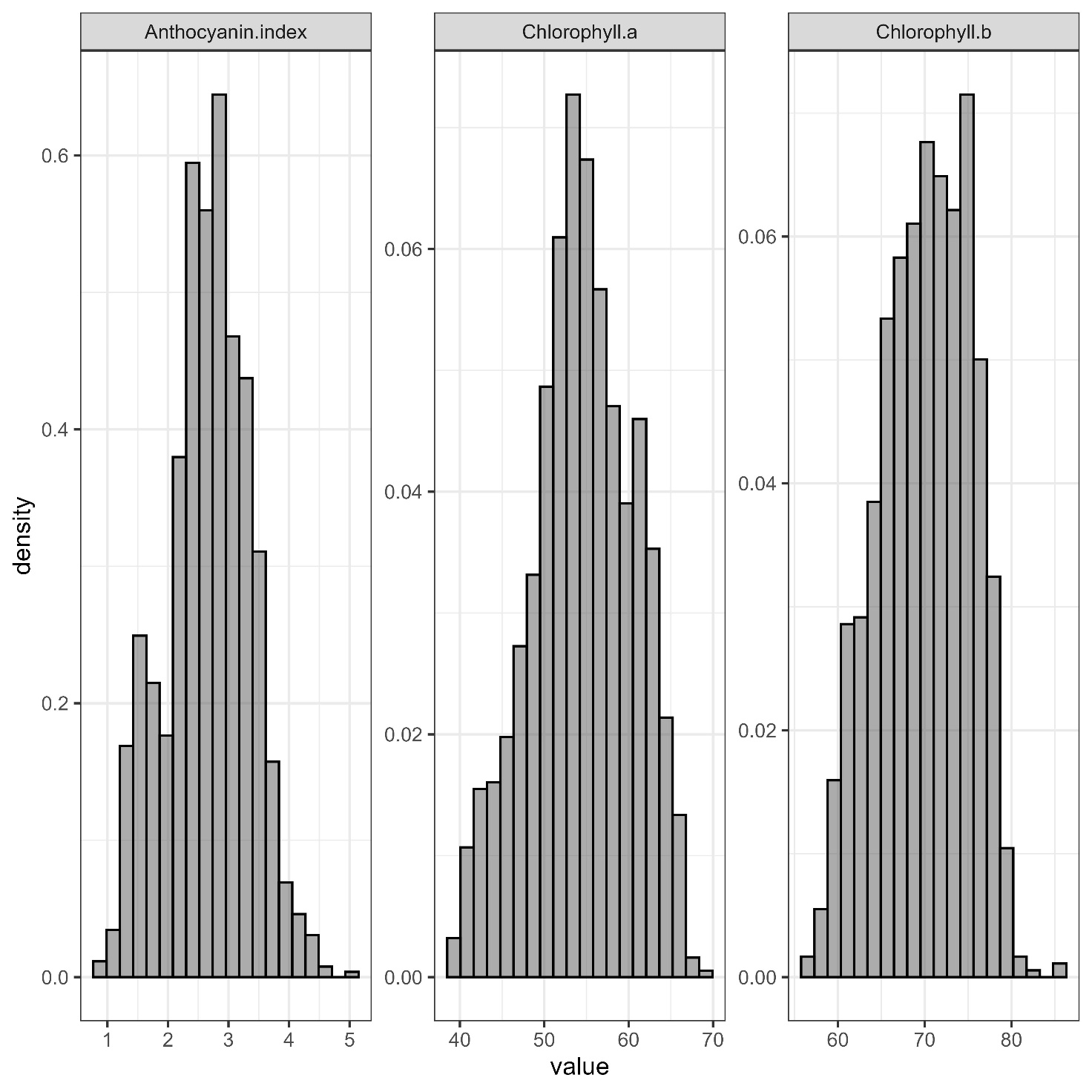


**Figure 2**. Density curve for the predictor variables anthocyanin index, chlorophyll *a* and chlorophyll *b* of peanut seeds that were used for the machine learning models obtained with the QDA method.

#
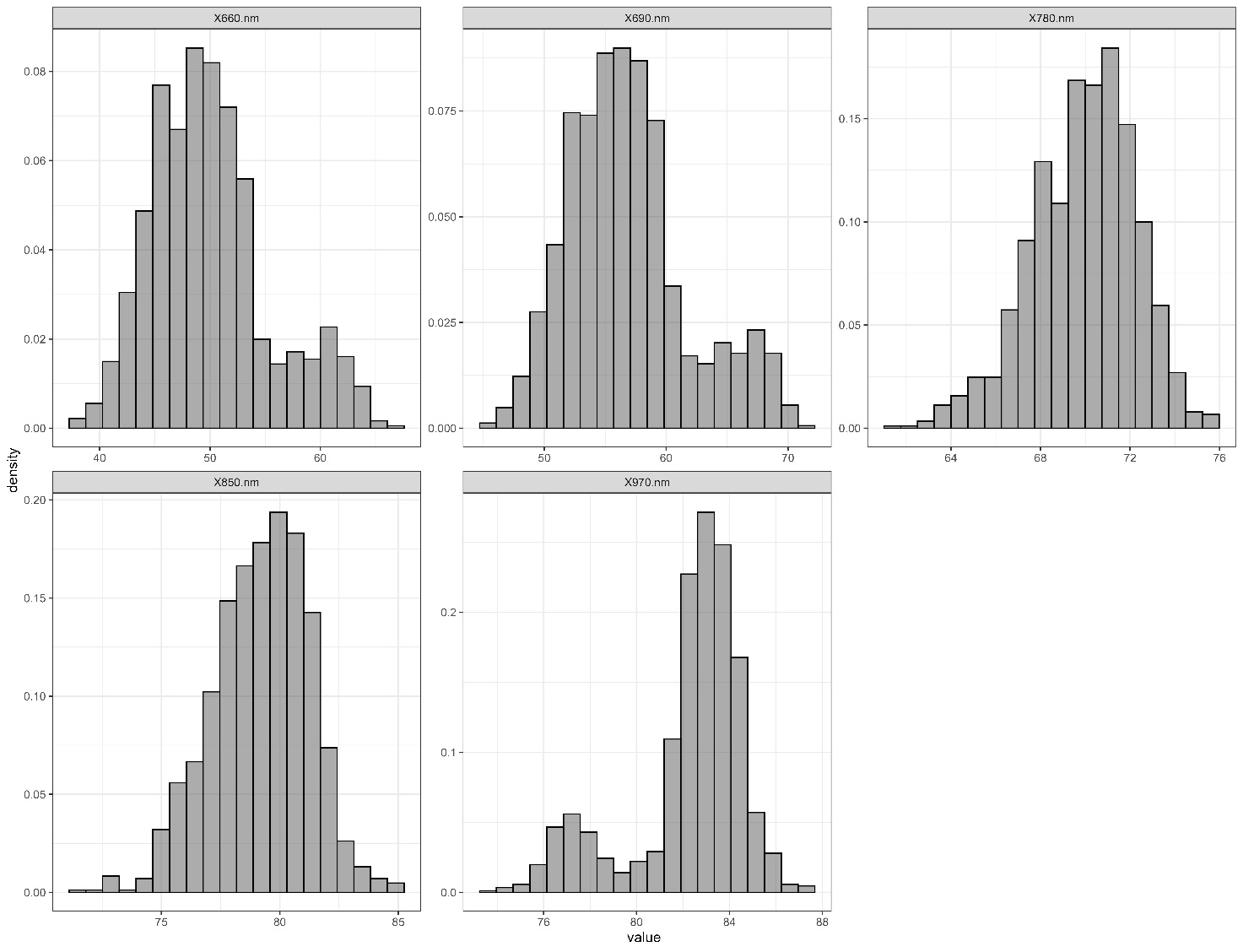


**Figure 3.** Density curve for the predictor variables 660, 690, 780, 850 and 970 nm of peanut seeds that were used for the machine learning models obtained with the QDA method.

# Libraries used

Clarke, W. R., Lachenbruch, P. A., and Broffitt, B. (1979). How non-normality affects the quadratic discriminant function. *Commun. Stat. - Theory Methods* 8, 1285–1301. doi:10.1080/03610927908827830.

Hastie, T., Tibshirani, R., and Friedman, J. H. (2009). *The Elements of Statistical Learning: Data Mining, Inference, and Prediction.* 2ed ed. Germany: Springer.
