# Supplementary material for: An Approach Using Emerging Optical Technologies and Artificial Intelligence Brings New Markers to Evaluate Peanut Seed Quality
Source: Front Plant Sci. 2022 Apr 14;13:849986. doi: 10.3389/fpls.2022.849986 (PMC9048030; doi:10.3389/fpls.2022.849986)
Supplement: Supplementary file 4 [file Data_Sheet_2.docx]

**Supplementary methodology 2**

**Analysis of peanut seed reflectance data - interactive process**

The iterative process aimed to select the predictor variables (bands) that presented the highest measure of accuracy, of the QDA model, in relation to the response variable (seedlots). In this way, computational procedures were performed that will be described below:

1. First, a cross-validation was carried out, partitioning the reflectance data obtained into 30% for testing and 70% for training;
2. The structure of the QDA model was defined, in which the qualitative information (seed lot) was considered as the model's response; the 19 bands (from 365 to 970 nm) were the numerical measurements considered as predictor variables;
3. Through an iterative process (for loop) all possible models were adjusted for each combination of 5 predictor variables of the 19 quantitative variables ($\left( \begin{aligned} 19 \\ 5 \end{aligned} \right)$ = 11628).
4. For each model, the accuracy measure of the models obtained for the test data was removed.
5. Finally, based on the ranking of these accuracy measures, the model with the highest accuracy measure was selected. The decreasing order of accuracy presented, gathers only the first 20 models.

# Libraries used

# All analyzes were performed using the R 4.1.0 language (2021-05-18), with the RStudio 1.4.1717 integration platform. The QDA analysis (quadratic discriminant analysis) was performed with the MASS library (Venables, 2002) with the MASS::qda() function, and the results of the confusion matrix and accuracy measure were collected by the library and caret:: confusionMatrix() (Kuhn, 2017).
