# Supplementary figures and images for: An Approach Using Emerging Optical Technologies and Artificial Intelligence Brings New Markers to Evaluate Peanut Seed Quality
Source: Front Plant Sci. 2022 Apr 14;13:849986. doi: 10.3389/fpls.2022.849986 (PMC9048030; doi:10.3389/fpls.2022.849986)

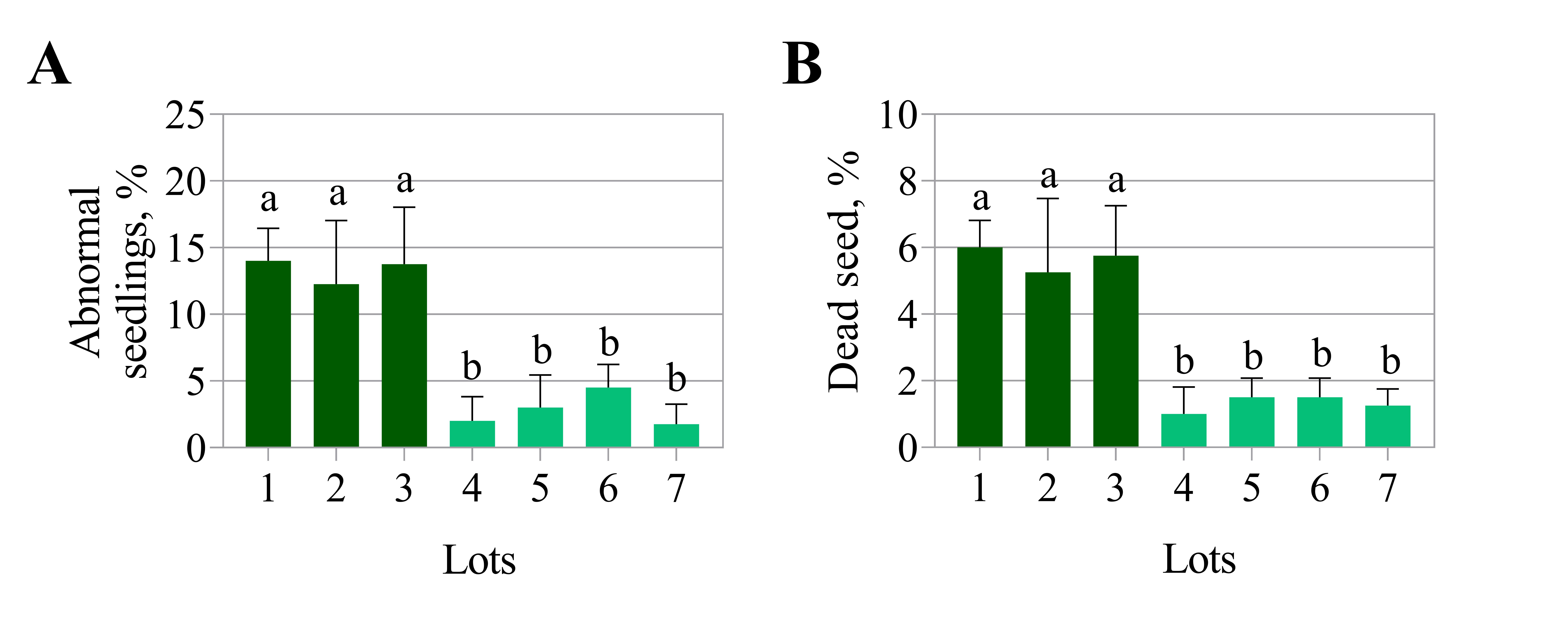

Supplement: Supplementary file 6 [file Image_2.JPEG]
